# Supplementary material for: An open-source framework for large-scale, flexible evaluation of biomedical text mining systems
Source: J Biomed Discov Collab. 2008 Jan 29;3:1. doi: 10.1186/1747-5333-3-1 (PMC2276192; doi:10.1186/1747-5333-3-1)
Supplement: Additional file 1 — Evaluation platform source code. The additional file contains the software evaluation framework discussed in this paper. The most current release can be downloaded from SourceForge.net [1]. See the accompanying README file for instructions on installation and use. Also included in the distribution is a collection of UIMA wrappers for some commonly used BioNLP tools and annotated corpora. [file 1747-5333-3-1-S1.zip › bionlp_uima_repository-1.0.1/docs/api/src-html/edu/uchsc/ccp/util/nlp/annotation/comparison/EntityPartsAnnotationComparator.html]

```
001    package edu.uchsc.ccp.util.nlp.annotation.comparison;
002    
003    import java.util.ArrayList;
004    import java.util.List;
005    
006    import edu.uchsc.ccp.util.nlp.GenericTextUtils;
007    import edu.uchsc.ccp.util.nlp.annotation.AnnotationSet;
008    import edu.uchsc.ccp.util.nlp.annotation.TextAnnotation;
009    import edu.uchsc.ccp.util.nlp.mention.ClassMention;
010    import edu.uchsc.ccp.util.nlp.mention.comparison.ClassMentionComparator;
011    import edu.uchsc.ccp.util.nlp.stats.PRFResult;
012    
013    /**
014     * This class implements the functionality of the "Protein Name Parts" comparison metric described in Olson et al...<br>
015     * The usefulness of this kind of comparison is limited to simple entity mentions, therefore, the mention comparison will compare the entire mention
016     * hierarchy, however, the entity parts computation will only take place on the base annotation span.<br>
017     * <br>
018     * For example, if for some reason you wanted to run the entity-parts metric on a group of transport annotations, where each transport mention has
019     * complex slot mentions (transported entities, source, destination, transport participants), then this method will<br>
020     * <ol>
021     * <li> find equivalent transport annotations using the SloppySpanComparator
022     * <li> if equivalent annotations are found, then the covered text for each annotation (and in this case, just for the transport annotation, not any
023     * of its slot fillers) will be tokenized on whitespace, and the entity-parts metric will be done using those tokens.
024     * </ol>
025     * 
026     * @author williamb
027     * 
028     */
029    public class EntityPartsAnnotationComparator extends AnnotationComparator {
030    
031        @Override
032        public PRFResult compare(List<TextAnnotation> goldTaList, List<TextAnnotation> testTaList) {
033    
034            List<TextAnnotation> goldAnnotationPieces = new ArrayList<TextAnnotation>();
035            List<TextAnnotation> testAnnotationPieces = new ArrayList<TextAnnotation>();
036    
037            /* for each gold annotation, split it and add its pieces to the pieces list */
038            for (TextAnnotation ta : goldTaList) {
039                List<TextAnnotation> pieces = tokenizeAnnotation(ta);
040                goldAnnotationPieces.addAll(pieces);
041            }
042    
043            /* for each test annotation, split it and add its pieces to the pieces list */
044            for (TextAnnotation ta : testTaList) {
045                List<TextAnnotation> pieces = tokenizeAnnotation(ta);
046                testAnnotationPieces.addAll(pieces);
047            }
048    
049            AnnotationComparator ac = new AnnotationComparator();
050            return ac.compare(goldAnnotationPieces, testAnnotationPieces);
051    
052        }
053    
054        /**
055         * Since this method returns an integer, it does not make sense to use it to compare the entity parts, however, in order to override the default
056         * compare() method of AnnotationComparator which employs the strict span comparator, we will implement this method to return 0 if the resulting F
057         * measure is 1.0, -1 otherwise.
058         */
059        @Override
060        public int compare(TextAnnotation goldTA, TextAnnotation testTA) {
061            List<TextAnnotation> goldTAList = new ArrayList<TextAnnotation>();
062            List<TextAnnotation> testTAList = new ArrayList<TextAnnotation>();
063    
064            goldTAList.add(goldTA);
065            testTAList.add(testTA);
066    
067            PRFResult prf = compare(goldTAList, testTAList);
068            if (prf.getFmeasure() == 1.0) {
069                return 0;
070            } else {
071                return -1;
072            }
073    
074        }
075    
076        /*
077         * Tokenize an annotation by whitespace.. this process removes any mention structure below the initial class mention.
078         * 
079         * Stopwords are not counted.
080         */
081        private static List<TextAnnotation> tokenizeAnnotation(TextAnnotation ta) {
082            List<TextAnnotation> annotationsToReturn = new ArrayList<TextAnnotation>();
083    
084            String coveredText = ta.getCoveredText();
085            String[] toks = coveredText.split(" ");
086    
087            int offset = ta.getAnnotationSpanStart();
088    
089            for (String tok : toks) {
090                if (!GenericTextUtils.isStopWord(tok)) {
091                    /* if it's not a stop word, then create a TextAnnotation */
092                    if (!tok.equals(" ")) {
093                        /* if it's not a space then create a TextAnnotation */
094                        TextAnnotation newAnnot = new TextAnnotation(offset, offset + tok.length(), tok, ta.getAnnotator(), new AnnotationSet(
095                                -1, "", ""), ta.getAnnotationID(), ta.getDocumentCollectionID(), ta.getDocumentID(), ta.getDocumentSectionID(),
096                                new ClassMention(ta.getClassMention().getMentionName()));
097                        annotationsToReturn.add(newAnnot);
098                    }
099                }
100                offset += (tok.length() + 1);
101            }
102            return annotationsToReturn;
103        }
104    
105        /**
106         * Overridden - note this ignores all extraneous input parameters and simple returns EntityPartsAnnotationComparator.compare(List<TextAnnotation>
107         * goldTaList, List<TextAnnotation> testTaList)
108         */
109        @Override
110        public PRFResult compare(List<TextAnnotation> goldTaList, List<TextAnnotation> testTaList, SpanComparator spanComparator,
111                ClassMentionComparator classMentionComparator, int maximumComparisonDepth) {
112            return compare(goldTaList, testTaList);
113        }
114    
115        /**
116         * Overridden - note this ignores all extraneous input parameters and simple returns EntityPartsAnnotationComparator.compare(List<TextAnnotation>
117         * goldTaList, List<TextAnnotation> testTaList)
118         */
119        @Override
120        public PRFResult compare(List<TextAnnotation> goldTaList, List<TextAnnotation> taList, SpanComparator spanComparator,
121                ClassMentionComparator classMentionComparator) {
122            return compare(goldTaList, taList);
123        }
124    
125        /**
126         * Overridden - note this ignores all extraneous input parameters and simple returns EntityPartsAnnotationComparator.compare(TextAnnotation ta1,
127         * TextAnnotation ta2)
128         */
129        @Override
130        public int compare(TextAnnotation ta1, TextAnnotation ta2, SpanComparator spanComparator,
131                ClassMentionComparator classMentionComparator, int maximumComparisonDepth) {
132            return compare(ta1, ta2);
133        }
134    
135        /**
136         * Overridden - note this ignores all extraneous input parameters and simple returns EntityPartsAnnotationComparator.compare(TextAnnotation ta1,
137         * TextAnnotation ta2)
138         */
139        @Override
140        public int compare(TextAnnotation ta1, TextAnnotation ta2, SpanComparator spanComparator, ClassMentionComparator classMentionComparator) {
141            return compare(ta1, ta2);
142        }
143    
144    }
```
